# Sex-Specific Features of Calcific Aortic Valve Disease

**DOI:** 10.3390/ijms21165620

**Published:** 2020-08-06

**Authors:** Volha I. Summerhill, Donato Moschetta, Alexander N. Orekhov, Paolo Poggio, Veronika A. Myasoedova

**Affiliations:** 1 Department of Basic Research, Institute for Atherosclerosis Research, Skolkovo Innovative Center, 121609 Moscow, Russia; a.h.opexob@gmail.com; 2 Unit for the Study of Aortic, Valvular and Coronary Pathologies, Monzino Cardiology Center IRCCS, 20138 Milan, Italy; donato.moschetta@ccfm.it (D.M.); paolo.poggio@ccfm.it (P.P.); veronika.myasoedova@ccfm.it (V.A.M.); 3 Department of Pharmacological and Biomolecular Sciences, The University of Milan, 20133 Milan, Italy; 4 Laboratory of Angiopathology, Institute of General Pathology and Pathophysiology, Russian Academy of Medical Sciences, 125315 Moscow, Russia

**Keywords:** calcific aortic valve disease, aortic valve calcification, aortic valve stenosis, sex differences, fibrosis, left ventricular hypertrophy

## Abstract

Calcific aortic valve disease (CAVD) is the most common valvular heart disease in developed countries predominantly affecting the elderly population therefore posing a large economic burden. It is a gradually progressive condition ranging from mild valve calcification and thickening, without the hemodynamic obstruction, to severe calcification impairing leaflet motion, known as aortic stenosis (AS). The progression of CAVD occurs over many years, and it is extremely variable among individuals. It is also associated with an increased risk of coronary events and mortality. The recent insights into the CAVD pathophysiology included an important role of sex. Accumulating evidence suggests that, in patients with CAVD, sex can determine important differences in the relationship between valvular calcification process, fibrosis, and aortic stenosis hemodynamic severity between men and women. Consequently, it has implications on the development of different valvular phenotypes, left ventricular hypertrophy, and cardiovascular outcomes in men and women. Along these lines, taking into account the sex-related differences in diagnosis, prognosis, and treatment outcomes is of profound importance. In this review, the sex-related differences in patients with CAVD, in terms of pathobiology, clinical phenotypes, and outcomes were discussed.

## 1. Introduction

Calcific aortic valve disease (CAVD) is the most frequent valvular heart disease in the Western world that, in the USA alone, affects 2.5% of the senior population (older than 65 years of age) accounting for 17,000 deaths annually [1]. The prevalence of CAVD increases with age and the male sex recognized as risk factor for aortic stenosis (AS), the end-stage of the disease [2]. The Multi-Ethnic Study of Atherosclerosis showed that the aortic valve calcification (AVC) incidence rate increased significantly with age and its risk of progression was associated with male sex and Agatston score at baseline [3]. A population-based study also reported higher prevalence of AVC in men versus women (33% versus 22%) which persisted after adjustment for age [4]. It was estimated that 12.4% (Europe) and 3.4% (North America) of the general elderly population (75 years and older) have symptomatic severe aortic stenosis (AS), the end stage of CAVD [5]. These figures are set to rise with current trends in the population demographics (i.e., ageing of the population) and the deficiency in effective prevention strategies [5]. The same study predicted that, by the year of 2050, there will be 2.1 million and 1.4 million patients with symptomatic severe aortic stenosis in Europe and North America, respectively. Moreover, the absolute number of patients with AS in Africa, Asia, and South America is also expected to increase with more pronounced demographic changes. The mortality rate is over 50% at 2 years in symptomatic AS patients unless the surgery of aortic-valve replacement is done promptly [6]. In 2010, 65,000 aortic valve replacement (AVR) procedures in total were performed in the United States, predominantly for AS and patients over 65 years of age were accountable for 70% of these procedures [7]. That underscored the high economic expenditure of health care in the ageing population.

The mechanisms controlling the initiation and progression of CAVD are not fully understood yet. The effect of sex, as an important modulator of pathological processes implicated in the development of CAVD, has emerged only recently and remains largely unexplored [8]. In this review, sex-related differences in patients with CAVD in terms of pathobiology, clinical phenotypes, and outcomes were discussed.

## 2. Calcific Aortic Valve Disease: Background

According to our current understanding, similarly to atherosclerosis, CAVD is a dynamic process with lipid accumulation, chronic inflammation, and active valve leaflet calcification. Calcification of aortic valves is the intrinsic process of valvular degeneration preceding AS which is initially mild or moderate, and eventually progressing to a severe degree with or without clinical symptoms. The progression of CAVD occurs over many years and is extremely variable amongst individuals. The end-stage of CAVD is characterized by blockage of left ventricular outflow tract, leading to a disturbance of cardiac output, diminished exercise capacity, secondary left ventricular hypertrophy (LVH) leading to heart failure, and mortality from major cardiovascular events. Clinical factors associated with CAVD are long-established and reflect those associated with coronary atherosclerosis [2]. Interestingly, coronary artery disease (CAD) is frequently found in subjects with AS. Low-density lipoprotein cholesterol (LDL-C) plays a major role in the cell signaling of vascular and valvular calcification. It was shown that calcification in both coronary artery and aortic valve advanced more rapidly in patients with higher LDL-C levels [9]. Moreover, lipoprotein(a), the main carrier of oxidized phospholipids is also associated with both CAVD and atherosclerosis supporting the important role of oxidative stress in the development of these processes [10,11]. Alongside the older age, studies demonstrated direct links between CAVD and male sex, hypertension, cigarette smoking, type II diabetes mellitus, and the metabolic syndrome [2,3]. The overlap in the clinical factors related to both atherosclerosis and CAVD as well as the correlation with the severity of coronary artery disease (CAD) and AVC are suggestive of the common developmental process. Nevertheless, it should be kept in mind that CAVD has unique features such as a calcium predominance and slower progression rate compared to atherosclerosis [12]. The cellular and molecular changes that occur during the onset and progression of CAVD have previously been described in detail by Towler et al. [13]. The earliest pathological change begins at the ventricular side of the valve endothelium with lipid accumulation and downward displacement and fragmentation of the subjacent elastic lamina. It is likely that endothelial dysfunction precedes these changes, since valve endothelial cells are profoundly involved in the initial resistance against metabolic, mechanical, and inflammatory damage [14]. Endothelial dysfunction, which is a hallmark of atherosclerotic process, was also found to be associated with aortic valve sclerosis, the initial stage of CAVD [15]. Endothelial damage is not only an initiating step but also a significant driving force in CAVD. Damage to the endothelium enables lipids to penetrate the valvular endothelium, their accumulation, and further increasing inflammation. The lipoproteins, such as low-density lipoprotein (LDL) and lipoprotein(a), undergo oxidation in the subendothelial layer and then taken up by macrophages that become foam cells. The oxidized lipids are extremely cytotoxic and capable of activating intense inflammation with subsequent calcification within the valve. An inflammatory infiltrate containing predominantly T-cells and macrophages is developed [16]. The T-cells and macrophages contribute to the increase of proinflammatory cytokines such as transforming growth factor–beta-1 (TGF-1β), tumor necrosis factor–alpha (TNF-α), and interleukin-1–beta (IL-1β) [17,18]. Moreover, valve endothelial cells subjected to these stimuli can contribute to CAVD progression through the process called endothelial–mesenchymal transition (EndMT) [19]. Inflammatory cells are also capable of inducing valve interstitial cell (VIC) differentiation into myofibroblast-like and osteoblastic-like cells leading the extracellular matrix aberrant remodeling and formation of calcium nodules [20]. The production of pro-inflammatory cytokine, such as IL-1β, is associated with the increased local production of matrix metalloproteinases (MMPs) and cellular proliferation in human aortic valves affected by calcific AS [21]. Secreted by myofibroblast-like cells and inflammatory cells, MMPs have an important role in the restructuring of the valve leaflet matrix. In combination with the action of MMPs and tissue inhibitors of metalloproteinases (TIMPs), disordered fibrous tissue accumulates within the valve [22]. Conversely, valve calcification is promoted by several pro-calcific pathways, including osteoprotegerin (OPG)/receptor activator of nuclear factor kappa B (RANK)/RANK ligand (RANKL) [23] and osteoblast differentiation signaling Lrp5/Wnt3 [24]. Subsequently, osteoblast-like cells control calcification of the valve by the regulatory process similar to skeletal bone formation, with the local production of various mediators, such as osteopontin, bone sialoprotein, osteocalcin, alkaline phosphatase (Alk-p), osteoblast-specific transcription factor Cbfa1, and bone morphogenic protein (BMP)-2 [20,25].

Accumulating evidence (presented in the next section) suggests that there are sex-related differences in the pathobiology of AS at valvular and ventricular levels. The combination of these two features are extremely important, since they determine the development of clinical phenotypes and subsequent adverse events that are characteristic of the advanced stages of the disease in both men and women.

## 3. Sex-Related Differences of Calcific Aortic Valve Stenosis: Pathobiology, Clinical Phenotypes, and Outcomes

### 3.1. Development of the Clinical Phenotypes

**Figure 1:**
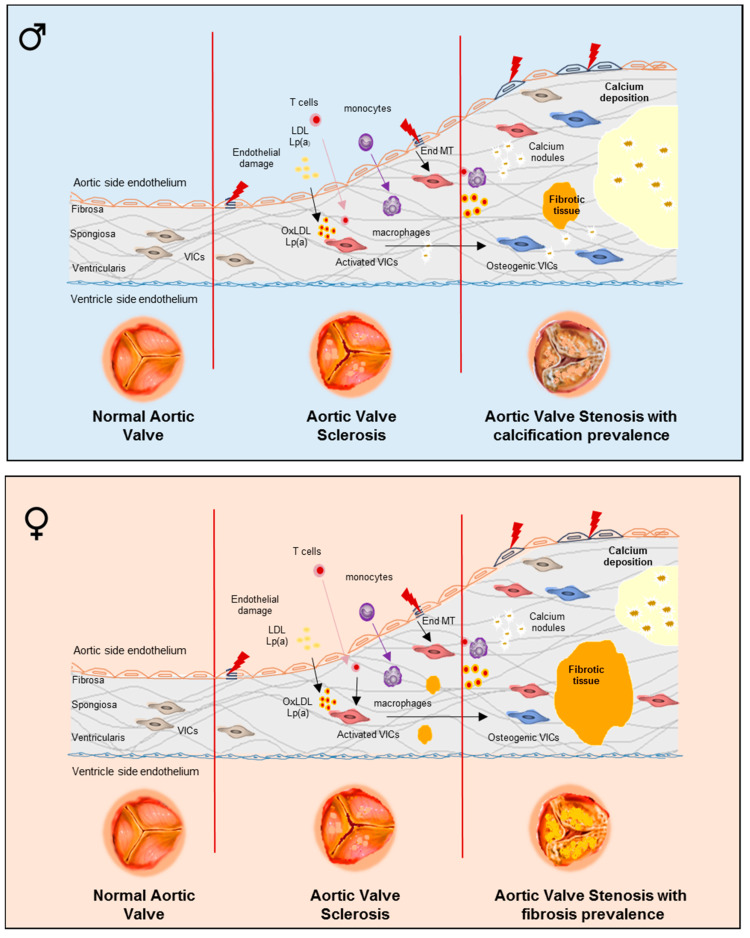
The development of valvular phenotypes in men and women with calcific aortic valve disease (CAVD) with underlying pathogenetic pathways. Summary image depicting CAVD progression in male and female. Normal aortic valve leaflet structure (fibrosa, spongiosa, and ventricular) with valvular interstitial cells (VICs) distributed in all three layers and a monolayer of valve endothelial cells (VECs) covering both sides. Aortic valve sclerosis (AVS) with normal hemodynamics (aortic jet velocity <2.5 m/s) is characterized by: (a) endothelial damage; (b) lipids infiltration and oxidation; (c) T-cell and monocyte infiltration and macrophage differentiation; (d) VIC activation; (e) endothelial to mesenchymal transition (EndMT); and (f) fibrosis and calcium nodule formation. Severe aortic valve stenosis (AS) has significant hemodynamic perturbations: (i) aortic jet velocity > 4.0 m/s; (ii) mean gradient > 40 mmHg; (iii) aortic valve area < 1.0 cm^2^; (iv) aortic valve area index to body surface < 0.60 cm^2^/m^2^. AS is characterized by: (a) endothelial damage; (b) lipids infiltration and oxidation; (c) T-cell and monocyte infiltration and macrophage differentiation; (d) activated and osteogenic VICs; (e) EndMT; and (f) with large calcium deposits prevalence in male and fibrosis prevalence in female.

Sex-related differences may be significant in the development of AVC and its transition to AS. Using cardiac multidetector computed tomography (MDCT), a large prospective, cross-sectional study of AVC, which included 665 patients, provided accurate and reproducible quantification of AVC load and the evaluation of aortic stenosis severity, in men and women, thus, presenting robust evidence that sex can influence not only the likelihood of CAVD development but also its nature [26]. The Agatston method was used to measure AVC exhibiting remarkable interscan, interobserver, and intra-observer reproducibility. The AVC load was lower in women than in men even after adjustment for their smaller body surface area and cross-sectional area of the aortic annulus, as typically observed in women. Moreover, the odds of high-AVC load were significantly higher in men versus women, while, overall, AVC showed strong associations with hemodynamic severity of AS both in men and women. Hence, for the same degree of AS, men had considerably greater valve calcification than women. Similarly, a prospective study including 646 AS patients with normal left ventricular ejection fraction showed that women develop hemodynamically more severe AS for the equal amount of AVC compared with men [27]. This study also showed that a substantial difference remained even after adjusting for body surface area and aortic annulus area. Therefore, the difference between men and women in the relationship between AVC loads and AS severity cannot be explained simply by the body constitution. This evidence is consistence with other studies that examined clinical, echocardiographic characteristics, and CT data of patients with AS and also indicated a significant difference in valvular calcium burden between men and women for the same degree of valvular stenosis [28,29]. Likewise, female sex was linked to borderline lower AVC progression; however, hemodynamic progression was not different between sexes [30]. These findings support the sex-related differences in the levels of AVC load required to reach hemodynamically severe AS. At that point, the question arose of whether, despite the potentially lower AVC progression, women develop similar hemodynamic progression due to the more fibrotic process than men. To confirm this, a recent prospective study by Simard et al. [31] on 125 patients with AS who underwent MDCT and Doppler echocardiography showed that the presence of higher levels of valvular fibrosis and dense connective tissue at the same degree of hemodynamic stenosis severity is more specific for women [31]. In addition, even after adjustment for age, body mass index, aortic valve calcification density, and aortic annulus diameter, female sex was associated with higher fibrosis score in AS valves. Moreover, in men, the degree of fibrosis clearly correlated with the amount of calcification. The results of this study on fibrosis of aortic valves were based on robust histological analyses carried out in corresponding subsets of male and female patients. Therefore, it was suggested that fibrosis might be a more significant contributor to valvular dysfunction in women versus men, thus, advocating sex, as a great determinant of pathobiological processes essential for the development and progression of AS [31]. A limitation of this work is the absence of quantitative data further than analysis of histological staining describing fibrosis. However, it remains unclear whether the CAVD progression course is fundamentally different for men versus women or if it is similar but with different exit points for each sex (Figure 1).

To elucidate the possible differences during the progression of CAVD, several recent studies have been conducted. A recent study demonstrated that, compared to men, women with AS had more pronounced fibrotic remodeling, irrespective of the valve morphology or age of the patient [32]. The same study also showed that, in CAVD, AS hemodynamic severity may not be correlated to the degree of AVC in young women with BAV so that these women may have a hemodynamically severe AS with no or minimal AVC load. This age-associated difference was not observed in men. Therefore, AVC quantification should be interpreted with caution in young women with BAV. Moreover, the influence of age and aortic valve phenotype on the relationship between AVC and AS hemodynamic severity was evaluated in another study which obtained similar results [33]. Amongst younger patients with BAV, some had a hemodynamically significant AS with minimal AVC density; thus, AVC as a predictor of AS severity can serve merely in older patients with BAVs. Other authors suggested that male sex in both BAVs and TAVs can be associated with greater rates of osseous and/or chondromatous metaplasia in calcific aortic valve stenosis [34]. Their histological study of aortic valves showed that men had a higher prevalence of metaplasia in both BAVs (33.5% versus 22.3%) and TAVs (13.8% versus 8.6%) compared to women. In addition, the occurrence of metaplasia was registered at younger age in patients with BAVs, providing another piece of evidence that aortic valve mineralization process is sex-specific and can be influenced by age and aortic valve structure.

Furthermore, qualitative assessment of AVC load may be of significant prognostic importance. The level of valvular calcification is the most valuable independent predictor of the disease progression in asymptomatic individuals [35]. It was shown that severe AVC density, defined according to sex, holds similar independent prognostic value for both men and women and in all possible AS sub-groups. Indeed, in severe AS, a high AVC load negatively correlated with clinical outcomes. As scored by echocardiography, moderate/severe AVC was associated with higher cardiovascular morbidity in both sexes and with higher all-cause mortality in men [29]. Additionally, the large-scale international registry study revealed that AVC load measured by MDCT can provide significant prognostic value for survival in patients with AS [36]. In this respect, AVC measurement by MDCT should be considered for not only diagnostic purposes but also risk prediction in patients with AS. Hence, when evaluating CAVD patients, the AVC load as well as the sex should be taken into account to ensure the development of sex-specific clinical approaches [8].

**Figure 2:**
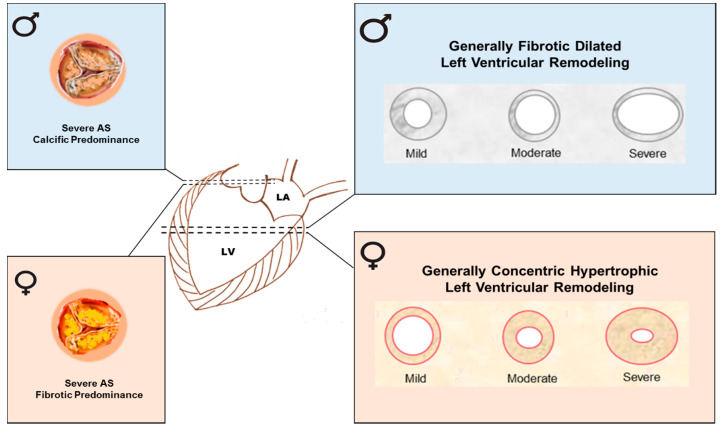
Sex-dependent differences in LVH phenotypes in men and women. Summary image depicting the effect of severe AS in men and women on LVH progression. LVH might correlate with severity of AS. Left ventricular remodeling and diffuse interstitial fibrosis increase during the AS progression. For a similar severity of AS, men show more advanced LV remodeling with larger indexed volumes, mass, mass/volume, lower ejection fraction, and more focal fibrosis, while women show higher prevalence of normal geometry or concentric remodeling with higher ejection fraction but relatively higher wall thickness.

Of note, CAVD patients present sex-related differences also at the ventricular level. In patients with severe AS, excessive pressure causes cardiac hypertrophy and fibrosis, in particular LVH which can later lead to heart failure. In the 1990s, it was established that, in elderly patients, LVH develops in a sex-specific fashion [37,38,39]. The studies indicated that, in general, men develop a fibrotic, dilated cardiomyopathy, whereas more concentric and hypertrophic changes in the myocardium with ventricular dilatation and failure, taking place at advanced stages of CAVD, occur in women (Figure 2). These studies were also confirmed by several recent ones [40,41].

Besides, higher transvalvular gradients, greater relative wall thickness, and a better systolic function were found in women [38]. Therefore, women more frequently develop heart failure with ejection fraction maintained, whereas, in general, heart failure in men is accompanied by a reduced ejection fraction [42]. Accordingly, at similarly reduced systolic function, better outcomes were reported in women with heart failure [40]. The abovementioned studies showed that, at equal valve severity, the left ventricular response to AS is more maladaptive in men than in women. Discordances in the development, progression, and outcome of pressure overload induced LVH between men and women can be determined by different molecular mechanisms active between the sexes. Transcriptome analysis revealed that maladaptive left ventricular remodeling can be associated with more pronounced activation of pro-fibrotic and inflammatory markers in men; while fibrosis-related and inflammatory pathways are suppressed in women [43]. In particular, ECM- and fibrosis-related genes had a different extent of regulation between the sexes. In consequence, myofibroblasts activating ECM–receptor interaction, renin-angiotensin system, and TGF-β signaling pathways were activated only in men. Furthermore, the expression of ECM proteins, such as periostin and collagen type I and III, were upregulated in the hearts of men with AS. Thus, the more pronounced activation of pro-fibrotic molecular mediators denotes that excessive fibrosis and ECM remodeling occur in male hearts, contributing to severe dilation of the left ventricle and impaired function. It is still unknown if the sex-related LVH-activated pathways are a direct effect of the different sex-related activated pathways within the aortic valve leaflets. Thus, further studies aiming to unveil the link between aortic valve and LVH pathological signaling are necessary. However, women and men with CAVD have different risk profiles which have a considerable impact on treatment outcomes and prognosis.

### 3.2. Post-Surgery Outcomes

It is well known that women can adapt to pressure overload differently from men. It was established that, despite more women than men with severe AS having LVH before AVR surgery, women more often experience faster regression of the LVH post-operatively [44]. Based on the above study that can be explained by the fact that due to the lower collagen I, III, and matrix metalloproteinase 2 gene expression, women have less fibrosis before the operation, leading to a quicker regression of LVH after surgery. Moreover, at the molecular level, reduced extracellular fibrosis can be associated with the protective effect of estrogen, since women have a higher number of estrogen receptors. In particular, a greater increase in the expression level of estrogen receptors beta (ERβ) was found in women patients with AS [45], while estrogen receptors alpha (ERα) are upregulated by pressure overload in both men and in women to the same extent [46]. The following cardioprotective actions of estrogen were extensively reviewed, and the major effects were the increment of angiogenesis and vasodilation and the decrease of the production of reactive oxygen species, oxidative stress, and fibrosis [47]. By these mechanisms, estrogen limits cardiac remodeling and reduces heart hypertrophy supporting the data indicating that females have a lower incidence of cardiovascular disease as compared to age-matched males [48,49,50]. Some animal studies exploring the cardioprotective effects of estrogens are presented in the next section.

A quicker LVH regression after transcutaneous aortic valve replacement (TAVR) in women, compared to men, was also observed in the latest study, indicating female sex as an independent predictor of favorable left ventricular remodeling post-operatively [51]. Moreover, the recent prospective Placement of Aortic Transcatheter Valve (PARTNER) clinical trial study reported both superior LVH regression and cardiovascular outcomes in elderly women, compared with men that had undergone TAVR [52]. This study demonstrated that after adjusting for known confounding variables, women had a lower rate (by over 50%) of ischemic cardiovascular events, such as stroke and coronary artery bypass surgery as well as lower total mortality by 31% during a 4-year median follow-up period. Notably, this was observed despite more severe AS based on echocardiographic indices and higher incidence of hypertension recorded in women, both common factors associated with worst prognosis in AS patients [53,54]. Similarly, another study, which included 379 patients with severe AS and with no prior atrial fibrillation, who underwent isolated AVR surgery, reported sex-related differences in factors linked to post-operative atrial fibrillation and its impact on mortality, with an increased risk of death observed in men but not in women [55]. Moreover, at 4-year follow-up, post-operative atrial fibrillation was associated with the increased risk of death only in men. There are other important factors associated with more favorable outcomes in women after TAVR. For example, women have lower incidence of baseline comorbidities, such as CAD, diabetes, history of previous myocardial infarction, and peripheral vascular disease [56]. This leads to lower pre-operative risk scores in women undergoing TAVR, in comparison to men. Indeed, the presence of residual aortic regurgitation, the important determinant of post-TAVR outcomes, is less common in women than in men [57]. It was found that a larger aortic annulus diameter in men is a driving factor of higher rates of moderate-to-severe aortic regurgitation after TAVR [58]. The impact of transvalvular flow, gradient, and sex, and the relationship among these parameters on mortality following AVR in patients with severe AS were also explored [59]. These findings highlight the importance of the sex-specific approach in clinical risk assessment in patients with AS and requiring either surgical AVR or TAVR. Indeed, a multivariable analysis considered female sex as an independent predictor of improved survival in patients older than 79 years of age [60]. However, several studies, evaluating sex differential impact on outcomes of AVR, obtained conflicting results [61,62,63]. Large-scale clinical studies are required to further assess sex-related differences in clinical outcomes following AVR and TAVR.

Thus, the data available indicated that, in patients with CAVD, sex could determine important differences in the relationship between development and progression of valvular calcification processes, fibrosis, and AS hemodynamic severity between men and women. Consequently, sex has implications on the development of different valvular phenotypes, LVH, and eventually cardiovascular outcomes.

## 4. The Experimental Evidence Indicating Sex-Related Differences in CAVD and LVH

The biological phenomena eliciting sex differences in the aortic valve and left ventricle are complex and unclear. Studies in vitro, ex vivo, and in vivo continue to identify potential complex-regulated mechanisms that may help to elucidate the impact of sex on the function of the aortic valve and left ventricular response to valve disease at the cellular and molecular level. Despite the fact that animal models were found to be particularly useful to study pressure overload by banding either the ascending or transverse aorta [64], CAVD research is lacking sufficient animal models. This might be due to the anatomical and pathophysiological differences between animal and human aortic valves. In addition, it is challenging to mimic CAVD in animals, as there is significant heterogeneity in the severity and the rate of progression of the disease in humans. Overall, the use of in vitro models is more advantageous due to the shortened experiment time and superior control over multiple variables.

### 4.1. Human Studies

Several intrinsic biological mechanisms were suggested that might help to clarify the difference in AVC load between men and women. Some authors reported a reduced calcification potential in human female VICs, compared to male ones (calcification induced by interferon-α) and sex-specific mechanisms involving apoptosis, such as phosphatidylinositol 3 kinase (PI3K)/Akt/cell signaling survival pathway [65]. Moreover, delayed kinetics in calcification progression and upregulation of matrix-Gla protein, the inhibitor of vascular mineralization, was found in female valves, as well as significantly higher basal expression levels of anti-apoptotic gene B-cell lymphoma (BCL)-2 were observed in both aortic valve tissue and VICs from females [65]. The same authors speculated that the differences in the secretion of IL-6, BMP-2, and MMP-1 might also be accountable for the lower calcification level in female aortic valves. Moreover, pro-angiogenic, pro-inflammatory, and pro-calcific effects of interferon-gamma (IFN-γ) in human VICs were found to be more prominent in male VICs than in female ones [66]. The results of this study disclosed a more robust activation of extracellular signal-regulated kinases (ERK) and hypoxia inducible factor-alpha (HIF-1α) via signal transducer and activator of transcription-1 (STAT-1) pathways in male aortic VICs. As mentioned above, both inflammatory and fibrotic responses in cardiac remodeling are less pronounced in women, compared to men. A study revealed molecular determinants of sex-dependent regulation of fibrosis and inflammation in human left ventricular remodeling under pressure overload [43]. In particular, in women undergoing pressure overload-induced left ventricular adverse remodeling, the JAK-STAT pathway-encoding genes were significantly downregulated. Women also showed a reduced expression of pro-fibrotic TGF-β and a lower inflammatory response.

### 4.2. Animal Studies

In in vitro culture of sex-separated VIC populations, isolated from healthy pigs, revealed that there were cellular-scale differences between healthy male and female porcine VICs [67]. Analysis of global gene expression profiles between healthy male and female VICs showed that several well-known CAVD-related biological pathways and processes may be intrinsically upregulated or primed to stimulation in male VICs, compared to female VICs. These pathways and processes included angiogenesis, lipid accumulation, extracellular matrix reorganization, and mitogen-activated protein kinase (MAPK)/extracellular signal-regulated kinases-1/2 (ERK)-1/2 pathways. These data suggested that predisposition to CAVD can be sex-determined on the cellular level in healthy people and that could have profound clinical implications. Some of the pathways and processes, such as angiogenesis, lipid accumulation, MAPK/ERK-1/2 pathways, and inflammation, were identified in calcified stenotic human valves [68]. In particular, related to regulation of various cellular functions, such as proliferation, differentiation, migration, senescence, and apoptosis, MAPK/ERK pathway is involved in VIC calcification and osteoblastic differentiation [69,70]. The role of ERK pathways as important regulators of matrix mineralization processes was described [71]. High levels of ERK phosphorylation were detected in calcified and stenotic human valves [72]. In support of the hypothesis of early occurrence of sex-specific differences in CAVD pathogenesis, another study using VICs obtained from male and female rat and porcine aortic valves showed that there is a sex-related difference in the events associated with osteogenic differentiation of the aortic VICs, where male VICs are more prone to calcification than female ones [73]. Human VICs undergo osteoblastic differentiation in response to specific mediators such as BMPs and TGF-β [74]. Moreover, it was established that a large number of genes that were assigned to the predominant early response to pressure overload was differentially regulated in male and female mice models before the occurrence of apparent sex-specific morphological or functional changes [75]. In this study, the network of genes encoding mitochondrial function and fatty acid oxidation was less downregulated in female hearts, while a relative upregulation of gene networks of matrix remodeling and ribosomes was found in male hearts. In particular, fatty acid oxidation is the important molecular pathway implicated into myocardial energetic metabolism, the impairment of which leads to cardiac failure [76]. Thus, these results indicated a better adaptation of energy metabolism in female hearts, in contrast to greater stimulation of matrix turnover and synthesis of ribosomal proteins in male hearts, the key mediators of sex-dependent remodeling in early stages of pressure overload. Hence, revealing regulatory genetic pathways of these processes may explain the relative protection of the female hearts in the early response to pressure overload and provide potential molecular grounds underlying the sex-specific functional changes seen at late stages of pressure overload and LVH in men and women.

Some evidence emerged indicating several signaling pathways implicated in mediating sex differences detected in response to pressure overload. In this respect, using transverse aortic constriction (TAC) murine models, a study revealed the importance of the calcium-calmodulin-dependent kinase II-myocyte enhancer factor 2 (CaMKII-MEF2) signaling pathway, as following the pressure overload, CaMK-phosphatase compartmentalization differed between sexes (inhibited in males) resulting in differences in MEF2 activation (inhibited in males), which can stimulate cardiac hypertrophy [77]. One more study identified another potential signaling mechanism mediating sex-related dimorphisms associated with LVH caused by pressure overload [78]. Using pressure overload rat models (male and female TAC Wistar rats), it was shown that the respective expression of constitutive nitric oxide synthase (NOS) and caveolins, the signal transduction proteins, is differentially regulated according to sex, corroborating the sex-related difference in cardiac adaptation to pressure overload [78]. In this study, male rats with TAC presented with early signs of cardiac dysfunction, whereas female rats with TAC had higher LVH, according to the echocardiographic and LV end-diastolic pressure measurements. Moreover, it was shown that the pattern of NOS expression and/or activity is regulated according to sex and cardiac functional condition [78]. In response to pressure overload, cardiac NOS1 expression was promptly induced and subsequently stable in males, whereas it was postponed in females. Whether these sex differences arise from intrinsic myocardial molecular adaptations to pressure overload remains unknown. Nitric oxide bioavailability, which is involved in the development of LVH and in the progression to heart failure, was proposed as a potential mechanism involved in these sex differences [79,80].

Besides, a sex-specific microRNA-29b (miR-29b) dysregulation was observed in mice subjected to TAC, pointing out a sex-influenced left ventricle remodeling pattern [81]. This study provided the evidence demonstrating that sex differences in left ventricular remodeling (molecular, structural, and morpho-functional) involve sex-specific regulation of miR-29b in mice with TAC and under the clinical AS conditions. The upregulation of miR-29b was identified in TAC female mice, while it was downregulated in TAC-males which was associated with a predominantly fibrotic and hypertrophic pattern of left ventricular remodeling, respectively. Remarkably, most of miR-29b-associated sexually dimorphic features of left ventricular remodeling detected in mice were observed in controls and patients with AS; therefore, the murine models provided new translational insights into CAVD pathogenesis. In addition, the preoperative plasma expression levels of miR-29b correlated with the severity of LVH in women with AS. The important role of other RNAs in some of the key processes responsible for CAVD progression, including phenotypic alterations of valvular cells and subsequent maladaptive left ventricular remodeling, was extensively reviewed by Gošev et al. [82].

Furthermore, there are other molecules, such as sarcoplasmic reticulum Ca^++^-adenosine triphosphatase, β-cardiac myosin heavy chain, and atrial natriuretic factor (ANF), where expression levels may determine sex-related differences in molecular remodeling in pressure overload hypertrophy. Investigating the left ventricular contractile reserve in the isolated heart in male and female Wistar rats with the use of TAC as a model of pressure overload, messenger ribonucleic acid (mRNA) expression levels of sarcoplasmic reticulum Ca^++^-adenosine triphosphatase were found to be depressed in male rats but not in female rats as compared with control rats [83]. Therefore, despite a similar level of LVH and systolic wall stress, female hearts maintained contractile reserve, whereas male hearts had weakened contractile reserve. The greater upregulation of β-cardiac myosin heavy chain and mRNA of ANF in male than in female ventricular myocardium was also observed in this animal model providing additional evidence indicating the reduction in contractile reserve in male LVH rats. This study revealed that there are remarkable sex-related differences in the expression levels of genes that play essential roles in cardiac calcium regeneration and contractile function at an early stage of chronic pressure overload.

Finally, several animal studies examined the effects of estrogen on cardiomyocyte hypertrophy proposing potential molecular mechanisms of sex-dependent ventricular responses. It was found that estrogen can attenuate the hypertrophic response to pressure overload in mice hindering the increased phosphorylation of p38-mitogen-activated protein kinase (p38-MAPK) and via increasing ANF expression [84]. Another study also demonstrated that estrogen can exhibit significant anti-hypertrophic effects on ventricular myocytes via transactivation of ANF [85]. Moreover, using aromatase knockout mice, which do not produce estrogens to both pathologic and physiologic stimuli, it was shown that, in women, estrogen may have an antihypertrophic effect on cardiac hypertrophy through multiple signaling pathways including phosphatidylinositol 3-kinase/Akt signaling and the β-catenin pathways [86]. It is well known that premenopausal women have greater cardiac Akt activity, compared to men or post-menopausal women with consequent greater antihypertrophic effects [87]. Estrogen receptors also mediate sex-specific anti-hypertrophic effects by different molecular mechanisms. The protective role of nongenomic ERα signaling in the aged female rat heart was demonstrated via a protein kinase Cε-dependent mechanism [88]. Particularly, acute ERα activation can lead to a reduction of ischemic-reperfusion injury and necrotic and/or apoptotic cell death in the aged female heart and that might contribute to explain sex differences in myocardial remodeling. Moreover, in female mice deficient in either ERα or ERβ, the hearts of ERβ-deficient knockout (βERKO) mice exhibited a more pronounced response to aortic banding, whereas αERKO mice were indistinguishable from wild-type mice, proposing that ERβ signaling is important in mediating the cardio-protection seen in normal females [89,90]. In agreement with these studies, the important role of ERβ in myocardial adaptation was also reported by other papers [91,92]. Using TAC mice, it was demonstrated that both female sex and ERβ can diminish the development of fibrosis and apoptosis, hence, delaying the progression to heart failure [91]. Besides, the administration of estrogen inhibited angiotensin II-induced cardiac fibrosis by ERβ signaling that diminished production of matrix metalloproteinase, collagens I, and III in female mice that underwent angiotensin II-induced cardiac hypertrophy [92].

Taken together, the experimental evidence discussed supports the important role of a number of molecular mechanisms implicated in CAVD and the development of LVH in men and women (Table 1). Evidently, they contribute to sex-specific phenotypic changes of aortic valve cells and regulate key processes underlying different patterns of valvular and left ventricular remodeling, including calcification, fibrosis, apoptosis, and inflammation. Collectively, the experimental studies provided a foundation for the discovery of pathophysiological mechanisms and novel molecular targets rendering sex as an important determining factor for the development of CAVD and LVH.

**Figure 3:**
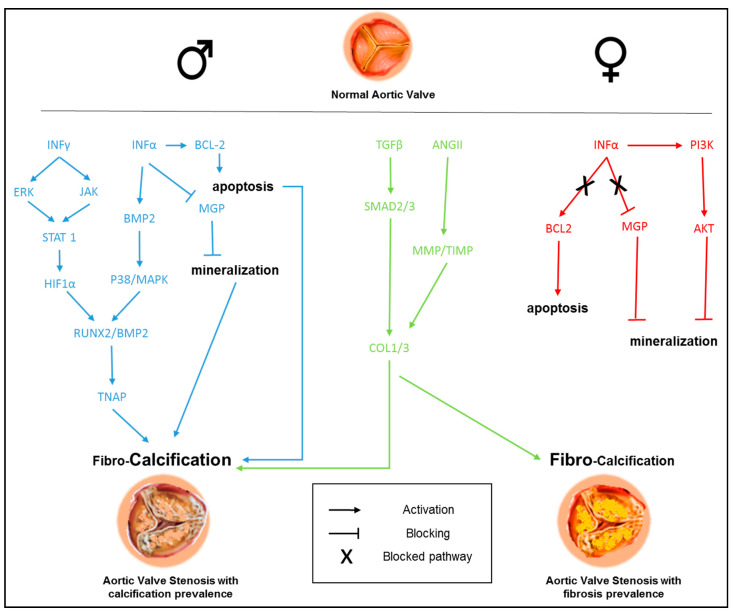
Sex-related signaling pathways in aortic valve fibro-calcification. The diagram represents the known sex differences observed in the signaling pathways implicated in calcific aortic valve disease (CAVD) pathogenesis and progression. Blue lines represent the male specific signaling pathway, red lines represent the female specific signaling pathways, while green lines represent generic signaling pathways.

A mechanism diagram covering the sex differences observed in the signaling pathways implicated in CAVD pathogenesis is presented in Figure 3.

## 5. Conclusions

Accumulating evidence suggests that sex is an important biological variable in CAVD pathogenesis. It could determine important differences in the relationship between development and progression of valvular calcification processes, fibrosis, and AS hemodynamic severity in men and women. Consequently, it has implications for the development of different valvular phenotypes, LVH, and cardiovascular outcomes in men and women. Along these lines, taking into account sex-related differences in diagnosis, treatment outcomes, and the prognosis is of deep importance. A better understanding of the different ways of CAVD development and progression in men and women might help the identification of novel beneficial sex-tailored prognostic/diagnostic strategies and therapeutic approaches for this clinically important disease. In particular, there is a great need in effective pharmacotherapies to treat those at high-risk for CAVD clinical progression because it might reduce or completely eliminate the necessity for invasive cardiac surgery and reduce the healthcare cost.

## Figures and Tables

**Table 1:** Overview of sex differences in the experimental models of CAVD and LVH.

Experimental Model			Experiment Description/Molecular Pathway	Tested in Females	Tested in Males	References
In Vitro	Ex Vivo	Animal Model				
Human aortic VICs			IFN-α–mediated inflammation and calcification		Observed to a higher degree	[65]
Human aortic VICs			PI3K/Akt/cell signaling survival pathway matrix-Gla protein expression BCL-2 gene expression	Upregulated		[65]
Human aortic VICs			IFN-γ-mediated pro-angiogenic, inflammatory, and calcific effects		Upregulated	[66]
	Human lateral LV wall tissue		JAK-STAT pathway	Downregulated		[43]
	Human lateral LV wall tissue		TGF-β expression	Downregulated		[43]
		TAC mice	LVH and heart failure	Induced	Induced	[64]
Porcine aortic VICs			Angiogenesis Lipid accumulation MAPK/ERK-1/2 pathway Inflammation		Upregulated	[67]
Rat and porcine aortic VICs			Osteogenic differentiation Calcification		Upregulated	[73]
	LV of TAC mice		Expression of genes encoding mitochondrial function and fatty acid oxidation	Upregulated		[75]
	LV of TAC mice		Expression of genes encoding ribosomal protein synthesis and extracellular matrix remodeling		Upregulated	[75]
	TAC B6D1/F1 mice		CaMKII-MEF2 pathway mediating cardiac response to PO	Upregulated		[77]
		TAC Wistar rats	LVH	Induced		[78]
		TAC Wistar rats	Cardiac dysfunction		Observed	[78]
	Whole LV extracts obtained from TAC Wistar rats		Cardiac NOS1 expression and activity associated with LVH caused by PO	Delayed	Rapidly induced	[78]
	LV fragments obtained from TAC Wistar rats		Caveolin-1 expression associated with LVH caused by PO		Downregulated	[78]
	LV samples obtained from TAC mice		miR-29b expression associated with LV remodeling pattern	Upregulated		[81]
	Isolated heart of TAC Wistar rats		Sarcoplasmic reticulum Ca++-adenosine triphosphatase expression (cardiac reserve in PO hypertrophy)		Downregulated	[83]
	Isolated heart of TAC Wistar rats		Expression of β-cardiac myosin heavy chain and ANF (cardiac reserve in PO hypertrophy		Upregulated	[83]
	Ovariectomized TAC mice		Estrogen-mediated anti-hypertrophic effect on PO hypertrophy via blocking of increased p38-MAPK phosphorylation and increasing ANF expression	Observed		[84]
Cardiomyocytes and fibroblasts obtained from hearts of Wistar–Kyoto rats			Estrogen-mediated anti-hypertrophic effect by inducing the ANF gene expression	Observed		[85]
		ArKO mice	Estrogen-mediated anti-hypertrophic effect through multiple signaling pathways	Observed		[86]

ANF, atrial natriuretic factor; ArKO mice, aromatase knockout mice; BCL-2, B-cell lymphoma 2 gene; CaMKII-MEF2, calcium-calmodulin-dependent kinase II-myocyte enhancer factor 2 pathway; ER-α, estrogen receptor alpha; ERβ KO mice, ERβ-deficient knockout mice; Gla, gamma-carboxyglutamic protein; IFN-α, interferon alpha; IFN-γ, interferon gamma; JAK-STAT, Janus kinase/signal transducer and activator of transcription; LV, left ventricle; LVH, left ventricular hypertrophy; MAPK/ERK-1/2, mitogen-activated protein kinase/extracellular signal-regulated kinases-1/2 pathway; miR-29b, microRNA-29b; p38-MAPK, p38-mitogen-activated protein kinase; PKCε, protein kinase Cε; PO, pressure overload; TAC, transverse aortic constriction; TGF-β, transforming growth factor-beta; TIMP-2, tissue inhibitor metalloproteinase-2; VICs, valvular interstitial cells.
